# Government health expenditures and health outcome nexus: a study on OECD countries

**DOI:** 10.3389/fpubh.2023.1123759

**Published:** 2023-04-17

**Authors:** Asim Anwar, Shabir Hyder, Norashidah Mohamed Nor, Mustafa Younis

**Affiliations:** ^1^Department of Management Sciences, COMSATS University Islamabad, Attock, Pakistan; ^2^School of Business and Economics, Universiti Putra Malaysia, Serdang, Selangor, Malaysia; ^3^Department of Health Policy and Management, School of Health Sciences, Jackson State University, Jackson, MS, United States

**Keywords:** health expenditures, infant mortality, life expectancy, air pollution, income, OECD countries

## Abstract

**Introduction:**

The consistent increase in health expenditures is an integral part of health policy. The aim of this study was to investigate the impact of health expenditures on health outcomes in the OECD countries.

**Method:**

We used the system generalized method of moments (GMM) for thirty eight OECD countries using panel data from 1996 to 2020.

**Results and discussion:**

The findings show that health expenditures have a negative impact on infant mortality while positive on life expectancy. The results further verify that the income measured as GDP, number of doctors, and air pollution has a negative effect on infant mortality, while these variables have a positive effect on life expectancy in the studied countries. The outcome of the study suggests that health expenditures need to be properly utilized and improvements can be made in the health policies to increase the investment in health technology. The government should also focus on measures like economic and environmental to have long-lasting health outcomes.

## Introduction

Healthcare is a persistent challenge for nations around the world. The growing economic and environmental challenges pose risks to the healthcare system (1–3). People suffer due to such risks and are prone to various diseases, including a child and maternal mortality, non-communicable diseases, infectious diseases, and lack of healthcare facilities (4, 5). According to research by the PEW research center, 85% of people consider the lack of healthcare facilities as a major problem in their respective healthcare systems (6). A good healthcare system is not only limited to treating diseases but also contributes to the economy (7, 8). Therefore, nations need to finance their healthcare system more effectively, which is a critical component of the health system (9–11).

Investment in healthcare is important for both short- and long-term benefits (12, 13). Good health, an important element of human capital, is considered one of the prerequisites for long-term sustainable economic development (14). The neoclassical growth model suggests healthy and educated human force increases the per capita income for individuals and their families which enhances the value of human life (7, 15, 16). Health expenditures can result in providing better health facilities and opportunities that strengthen human capital, leading to higher productivity and economic performance (8, 17). Increased public spending on curative care, emergency assistance, and vaccination and nutrition activities results in significant health outcomes in the form of reduced mortality (18). The literature has shown mixed results on the impact of healthcare expenditures on health outcomes. Some studies have shown that health expenditures positively contribute to health outcomes in terms of higher life expectancy and lower child mortality (14, 19, 20). For example, a study (14) found a positive impact of healthcare expenditures on health outcomes measured as life expectancy and maternal and infant mortality in the OECD countries. Similarly, examining 17 OECD countries for the period 1973 to 2000, a study by Kim and Lane (20) found a positive association between health spending and health outcome using infant and life expectancy at birth as health outcome indicators. Another study (1) emphasized that higher health spending improves life expectancy and reduces infant mortality. However, some studies (21, 22) found no relationship between health expenditures and mortality rate in European countries using Spearman's correlation method. A study (23) based on the review of the literature concluded that the relationship between healthcare expenditures and health outcome (life expectancy) is difficult to establish, while some researchers found an insignificant association between health expenditures and health status (24).

Governments around the globe acknowledge the importance of the healthcare system; therefore, health expenditures throughout the world have increased over time (25). Health expenditures are mostly financed through public taxation and are growing more than the global economy accounting for 10% of the world gross domestic product (GDP) (25). The average health expenditures as a share of GDP have increased from 7.8% in 2005 to 9.8% in 2020 in the OECD countries (26). The health expenditures in the United Kingdom, Germany, Portugal, Korea, and Italy have increased from 7.8, 8.4, 10.3, 9.7, 4.6, and 8.3 in 2005 to 9.8, 12.8, 12.5, 10.1, 8.4, and 9.7 in 2020, respectively (26). The swift increase in healthcare expenditures necessitates the need to examine whether such expenditures have really improved health outcomes in OECD countries (27, 28).

Thus, the contribution of this research in the health economics literature is manifold: first, to authenticate the relationship between health expenditures and health outcomes which so far has mixed results (29–31). Second, the OECD countries have the highest healthcare spending, i.e., almost 85% of the world's spending while its population is <20% of the world's total population (32). Therefore, it is necessary to study the impact of higher health expenditures on health outcomes in these countries as a test case for other countries to follow and improve their health infrastructure. Third, the recent studies on health expenditures and health outcomes in OECD countries used either cross-sectional data or the sample size does not cover all the OECD countries. For example, (14) using cross-sectional data for 1 year found that increased health spending improves life expectancy and reduces infant mortality in OECD countries. Christopoulos and Eleftheriou (19) using panel data for 29 OECD countries focused on the fiscal effects of health expenditures on health outcomes and found a significant impact of healthcare expenditures on increasing revenue. Aydan et al. (33), using panel data for OECD countries, focused on healthcare and social spending and found them to be important factors in explaining health outcomes. Therefore, it is important to comprehensively analyze the available data for all the OECD countries over time.

## Materials and methods

Various methods are used in the literature to explore the relationship between healthcare expenditures and health outcomes (34). Based on the availability and nature of data, the study used panel data estimation. Panel data estimation has various benefits: first, panel data control for the inter-country differences than cross-sectional or time series data; second, panel data even with unbalanced data provide reliable estimates; third, panel data provide higher degrees of freedom and sample variation (35, 36). Therefore, given these advantages, we use the health model following Rahman et al. (34), Novignon and Lawanson (37):


$$\begin{array}{l} y_{it}=H_{it}\;b\;+\;e_{t} \end{array}$$

where y_it_ is the dependent variable(s), i.e., infant mortality and life expectancy at birth. H is a vector of independent variables, i.e., government health expenditure, per capita GDP, number of doctors, and population of the country, while *b* is the vector of coefficient, *e* is the vector of stochastic terms, and *i* and *t* subscripts are used for individual country and time.

Higher government health expenditures would suggest more health facilities, provision of necessary medical equipment, and higher standards of hospitals. Therefore, these facilities are likely to improve the health of the citizens. This is also true for higher per capita GDP, where the higher income of the citizens not only increases the citizens' ability to spend more on treating their diseases but also helps them spend money on those activities which improve their health. One of the most important aspects of any medical infrastructure is the availability of doctors because ultimately it is the doctor that could use the medicine and health equipment to treat its patients. Therefore, doctors are the pillar of any medical system, and hence, we have included the number of doctors to signify the efficacy of the medical structure of the county. Air pollution is one of the main causes of mortality; therefore, we have used carbon dioxide emission as a proxy for air pollution.

Based on the aforementioned arguments, we have used the given econometric model having two equations with the following specifications:


$$\begin{array}{l} \text{I}{\text{F}}_{\text{it}}={\text{b}}_{0}+{\text{b}}_{1}\text{H}{\text{E}}_{\text{it}}+{\text{b}}_{2}\text{GD}{\text{P}}_{\text{it}}+{\text{b}}_{3}\text{Po}{\text{p}}_{\text{it}}+{\text{b}}_{4}\text{C}{\text{O}}_{2\text{it}}+\;{\text{e}}_{\text{it}}\\ \text{L}{\text{E}}_{\text{it}}={\text{b}}_{0}+{\text{b}}_{1}\text{H}{\text{E}}_{\text{it}}+{\text{b}}_{2}\text{GD}{\text{P}}_{\text{it}}+{\text{b}}_{3}\text{Po}{\text{p}}_{\text{it}}+{\text{b}}_{4}\text{C}{\text{O}}_{2\text{it}}+\;{\text{e}}_{\text{it}} \end{array}$$

where IF denotes infant mortality and is measured by the number of total deaths per one thousand live births, HE shows the government health expenditures per capita in US dollars, GDP measures the per capita GDP in US dollars, Pop shows the population, and CO_2_ measures the carbon dioxide emissions in tones per capita, while LE shows life expectancy at birth in years, *i* and *t* measure the usual cross-section and time, and *e* is the stochastic term.

The econometric model is estimated in double log form, i.e., both the dependent and independent variables are measured in natural logarithmic form; therefore, individual variables could be interpreted as elasticities. The study used the system generalized method of moments (GMM) for estimation. This is because in assessing the panel model estimation, usually, potential endogeneity issues arise because of unobserved heterogeneity and cross-sectional dependence. To overcome such issues, we used the system GMM. System GMM is also preferable because it requires the number of cross sections to be greater than the time period, which in our case is true; i.e., we have 38 countries, while the time period is 25 (38). In addition, our model has fewer instruments than the number of cross sections. Usually, the basic econometric methods to estimate panel data include panel fixed and random effect models. To select a better model out of the two methods, Durbin–Wu–Hausman test is used. Based on Durbin–Wu–Hausman test, we estimated the fixed effect model to compare our results with those of system GMM estimation.

The annual data for the study were obtained from the OECD dataset for the period from 1996 to 2020. There are 38 OECD member countries from different regions, and European member countries include Austria, Belgium, Czech Republic, Denmark, Estonia, Finland, France, Germany, Greece, Hungary, Iceland, Ireland, Italy, Latvia, Lithuania, Luxembourg, Netherlands, Norway, Poland, Portugal, Slovak Republic, Slovenia, Spain, Sweden, Switzerland, and the United Kingdom; from the United States of America, the member countries are Canada, Chile, Colombia, Mexico, Costa Rica, and the United States; from pacific, four member countries are Australia, Japan, Korea, and New Zealand; and from Middle East, there are two members, i.e., Israel and Turkey. Although most of the data were available, however, for some countries data were missing for some years, therefore our data set is unbalanced. The descriptive statistics for various variables are provided as follows:

As Table 1 indicates, the life expectancy is higher, i.e., on average OECD citizens live for around 79 years, and there is little variation within and among those countries, i.e., overall average age ranges from as low as 69 years to a maximum of around 85 years and this is also obvious from smaller values of standard deviation within and between countries. The lowest average is observed in Columbia and the highest in Japan. Similarly, the infant mortality overall average is low, i.e., only 5.5 children die out of 1,000 live births; however, in contrast to life expectancy, there is wide variation observed, i.e., the standard deviation value is much higher, i.e., around 4.5, given the overall average value of 5.5. This can similarly be observed in the overall range with a minimum of 0.7 to a maximum of around 43. This trend is observed within and between OECD countries. The highest infant mortality is observed in Turkey, i.e., 42.7, while the lowest is seen in the case of Iceland, i.e., 0.7.

**Table 1:** Descriptive statistics.

**Variable**		**Mean**	**Std Dev**	**Min**	**Max**
**Life expectancy**					
	Overall	78.68	3.23	69.1	84.7
	Between		2.77	72.97	82.58
	Within		1.76	73.63	83.20
**Infant mortality**					
	Overall	*5.56*	4.56	0.7	42.7
	Between		4.23	2.26	23.12
	Within		2.01	0.75	33.78
**Govt health expenditure**					
	Overall	2,112.4	1,332.20	159.78	10,052.33
	Between		1,047.75	427.82	4,447.91
	Within		849.41	−536.79	7,716.81
**GDP per capita**					
	Overall	32,209.5	16,836.24	5,807.335	117,721.2
	Between		13,765.32	10,422.87	82,180.15
	Within		9,938.14	−10,282.96	77,494.44
**Population**					
	Overall	33.47	54.36	0.26	331.50
	Between		54.92	0.31	302.61
	Within		3.85	0.25	62.35
**Doctors**					
	Overall	3.07	0.92	0.864	6.32
	Between		0.79	1.57	5.27
	Within		0.48	1.36	5.65
**CO**_2_					
	Overall	8.06	4.29	1.13	24.66
	Between		4.16	1.37	18.87
	Within		1.21	0.99	13.84

Most of the independent variables showed higher variation in overall values, and a similar trend is observed between and within the sample countries. For brevity, we will discuss only the overall values. For example, in the case of per capita government health expenditure, the overall average is around 2,100 US dollars. The overall wider variation is evident from the standard deviation value of around 1,300. The minimum overall value is as low as 160 dollars in the case of Turkey to as high as around 10,000 dollars in the case of the USA. Per capita GDP is higher among OECD countries; its overall average is around US $ 32,000. The wide variation is evident from the standard deviation value of around 16,000, which is almost half of the value of the average. The highest per capita GDP is observed in Luxemburg, i.e., around 11,800 while the lowest is observed in Latvia, i.e., around US $ 5,800. Overall, the average population in an OECD country is around 34 million; however, there is too much variation in the sample as the standard deviation value of 54 far exceeds the average. The minimum value is as low as 0.26 in the case of Iceland to a maximum of around 330 million in the case of the USA. The overall average number of doctors in an OECD country is around 3, although it has lower variation as compared to other variables, i.e., its value is 0.9. However, there is wide variation in terms of range, where the minimum number of doctors is as low as 0.86 in the case of Costa Rica and as high as 6.3 in the case of Greece. In the case of carbon dioxide emissions, the overall average is around 8. The wider variation, in this case, is shown by a standard deviation of around 4, i.e., almost half of the average value. This effect is also visible from the range of the values of this variable, where in the case of Costa Rica it has a minimum value of around 1 and the highest value is around 25 in the case of Luxemburg.

Although OECD countries are considered high-income countries with better infrastructure as compared to the rest of the world, however, among OECD countries there is wide variation as can be seen in the case of different variables, with the exception of life expectancy, where most of the countries show similar results.

## Results

Based on the results of the Durbin–Wu–Hausman test, we have chosen the panel fixed effect model. The results of the panel fixed effect model are shown in Table 2 and are discussed briefly. The results show that government health expenditures have a negative and significant effect on infant mortality while positive in the case of life expectancy. The results show that a 1% increase in government health expenditures will reduce infant mortality by 0.21% and improves life expectancy by 0.008%. Whereas, income and CO_2_ have a negative, the number of doctors and population has a positive impact on infant mortality and life expectancy.

**Table 2:** Results of the fixed and random effect model.

**Variables**	**Life expectancy**		**Infant mortality**	
	**FE**	**RE**	**FE**	**RE**
Constant	3.696^***^ (0.02)	3.722^***^ (0.01)	8.558^***^ (0.38)	8.840^***^ (0.32)
Govt. health expenditure	0.008^***^ (0.00)	0.0093^***^ (0.02)	−0.219^***^ (0.04)	−0.213^***^ (0.04)
GDP Per capita	0.0547^***^ (0.00)	0.0545^***^ (0.00)	−0.599^***^ (0.05)	−0.582^***^ (0.05)
Population	0.0141^***^ (0.00)	0.00545^**^ (0.00)	0.306^***^ (0.09)	0.117^***^ (0.03)
Doctors	0.003 (0.00)	0.00467^**^ (0.00)	0.104^**^ (0.04)	0.106^***^ (0.04)
CO_2_	−0.0005 (0.00)	−0.0026 (0.00)	−0.081^**^ (0.03)	−0.082^**^ (0.03)

^*^, ^**^, and ^***^ denote results significance at 10, 5, and 1%, respectively, while values in brackets denote standard errors, whereas HE denotes government health expenditures, POP denotes total population in million, CO_2_ shows carbon dioxide emission tones per capita, DOC shows the number of doctors per 1,000 inhabitants, GDP shows per capita GDP, and Lag shows the dependent variable's lagged values.

The results of the system GMM are shown in Table 3. We ran the regression on two different dependent variables, i.e., infant mortality and life expectancy while the independent variables remain the same in both models. In Table 3, column 1 represents the results of government health expenditures on infant mortality. The estimation results indicate a positive and significant impact of health expenditures on infant mortality in OECD countries. The result shows that a 1% increase in government health expenditures will reduce infant mortality by 0.28%. Air pollution is also considered to be an important factor affecting infant mortality; our results show the positive and significant effect of air pollution on mortality. It shows that if air pollution is increased by 1%, the mortality will increase by 0.58% in the studied countries.

**Table 3:** Results (system GMM).

**Variable**	**Infant mortality**	**Life expectancy**

	**1**	**2**
HE	−0.28^***^ (0.040)	0.0001 (0.001)
GDP	−0.29^***^ (−0.058)	0.010^***^ (0.002)
POP	0.71^***^ (0.071)	0.003 (0.004)
C0_2_	0.58 (0.127)	−0.006^***^ (−0.001)
DOC	−0.41 (−0.057)	0.004^*^ (0.002)
Lag	0.22^***^ (0.040)	0.79^***^ (0.028)
AR (1)	0.003	0.002
AR (2)	0.243	0.175
Hansen (OIR)	0.913	0.901

^*^, ^**^, and ^***^ denote results significance at 10, 5, and 1%, respectively, while values in brackets denote standard errors, whereas HE denotes government health expenditures, POP denotes total population in million, CO_2_ shows carbon dioxide emission tones per capita, DOC shows the number of doctors per 1,000 inhabitants, GDP shows per capita GDP, and Lag shows the dependent variable's lagged values.

Infant mortality is not only influenced by health expenditures and air pollution but also by other socio-economic factors like income and the number of doctors. Therefore, we added income and the number of doctors in our model and both of these variables showed a negative impact on mortality. The outcome shows that if we increase the number of doctors and income by 1%, infant mortality will decrease by 0.41 and 0.71%, respectively, whereas the result is positive in the case of the population.

To further validate the impact of health expenditures on health outcomes, we estimate the same model for health outcomes keeping life expectancy as a dependent variable. The independent variables remain the same. The results show a positive impact of health expenditures on life expectancy in the OECD countries. It shows that by increasing health expenditure by 1%, life expectancy will be increased by 0.001%. Whereas, the results of income and number of doctors show a positive impact highlighting a 1% increase in income and a number of doctors increase life expectancy by 0.05 and 0.003%, respectively, while a 1% increase in air pollution leads to a decrease in life expectancy by 0.004%.

## Discussion

The study aimed to analyze the role of government health expenditures on health outcomes in OECD countries using the system generalized method of moments (GMM). The results revealed a positive and significant association between health expenditures and health outcomes proxied by infant mortality and life expectancy at birth. The results are consistent with other studies that have shown similar results in the OECD countries (14, 19, 39). A study (14) found a positive impact of health expenditures on health outcomes in OECD countries while Akinci et al. (40) reported that increased health spending reduces infant mortality in MENA countries. In general, it is observed in countries, providing easy, affordable, and accessible health facilities, especially mother and child healthcare, immunization, and higher government funding for such programs result in lower infant and maternal mortality (41).

Similar to infant mortality, the study indicates that life expectancy also improves with the increase in health expenditures in the OECD countries which is in line with other studies showing a positive impact of health spending on life expectancy (14, 20). The studies (1, 14) found that life expectancy at birth increases with the increase in government health expenditures. The increase in government health expenditures improves the healthcare facilities which reduces the risk of illness through timely and effective utilization of healthcare facilities thus increasing the average life expectancy (14). The important role of government involvement in healthcare acquisition is widely accepted in the healthcare system (42). The government is in a better position to allocate resources to medical research and to develop infrastructure to achieve better health outcomes. The positive result of health spending, as indicated in our study as well as in other studies, translates into better health outcomes. In OECD countries, various government interventions such as the primary public service and the provision of free of cost or subsidized primary healthcare services to children result in better health outcomes (43). The average infant mortality rate in OECD countries stands at 4 per 1,000 live births, making notable progress in reducing the mortality rate by 40% over the last 18 years (44). Life expectancy in OECD countries has increased on average to 81 years, i.e. it has increased by 10 years in 2020 as compared to 1970 (45). Improvement in life expectancy could be attributed to better medical care provision; however, various aspects affecting adult health are also added to support the healthcare system. In this regard, the OECD countries including the United Kingdom, Australia, Turkey, Ireland, and New Zealand adopted comprehensive anti-tobacco policies, including regulation of tobacco use and public education, which were added to improve life expectancy (46). While some countries introduced a tax on the unhealthy diet to fight obesity and promote healthy lifestyles in OECD countries (46), circulatory diseases and cancer were the main reasons for mortality in the OECD countries. During the span from 2000 to 2019, ischemic heart diseases (IHDs) and strokes have decreased on average by 47 and 52% in OECD countries showing the importance of health spending in the studied countries (47). Our study also observed the positive impact of environmental quality on health outcomes. The results showed an increase in CO_2_ level increases infant mortality and reduces life expectancy. One might infer that an increase in emissions causes respiratory complications in adults and especially in children as children are more vulnerable to air pollution due to their higher air intake (48), while another research (49) stated that 96% of childhood mortality is instigated by air pollution due to lower respiratory infection. Moreover, exposure to ambient CO_2_ in the indoor environment has detrimental effects on the human body causing high blood pressure, heart diseases, and breathing problems. Our results are in line with other studies including (50–52). One interesting study (50) found a two-way causal relationship between CO_2_ emission and health expenditures in OECD countries.

The push for attaining higher economic growth causes air pollution to increase in the form of higher greenhouse gas emissions. Air pollution is a major risk factor for health causing respiratory, cardiovascular diseases, and lung cancer (50, 51). Although the CO2 emissions in the OECD countries are reduced by 9% in recent years, it was at their peak in the 2000's era (53). The effects of air pollution are long-lasting, thus, the reduction in air pollution in the OECD countries in recent years will not be fruitful at once.

The results also showed that an increase in the number of doctors improves the health status in the OECD countries. Various studies show similar results (54, 55). For example, one such study (56) noted that a 1% increase in the supply of medical doctors will decrease mortality by 0.08 per 100,000 population. The availability of quality medical staff is the key component of any healthcare system. In OECD countries, the number of doctors has increased over the years, and there were 3.5 doctors per 1,000 population on average in 2019 compared to 2.7 in 2000 (57). The other important variable, i.e., per capita income, shows a positive effect on life expectancy and a negative on mortality. Other studies also noted that income has a positive effect on life expectancy and a negative on mortality (48, 58, 59). Nations with a higher level of national income tend to spend more on health and support their people by providing better healthcare facilities thus increasing life expectancy and reducing mortality (48, 60). Moreover, individuals with higher income levels tend to be more health conscious and can spend more on their health thus reducing diseases and mortality (61).

This study used life expectancy and infant mortality as indicators for health outcomes; future studies may study other variables for health outcomes. Moreover, other socio-economic variables are also important to analyze that could improve health outcomes like education, income inequality, unemployment, and lifestyle. Therefore, future research needs to study these socio-economic variables for their impact on health outcomes. Moreover, such studies are also needed in the context of developing countries.

## Conclusion

Health expenditures act as an important pre-requisite for healthcare performance. The current study investigated the impact of health expenditures on health outcomes using infant mortality and life expectancy as proxies in the OECD countries. The study contributes to the body of literature by studying the impact of health expenditures and other socio-economic and environmental factors on health outcomes in the OECD countries. The results confirmed the negative impact of health expenditures on infant mortality and the positive on life expectancy in the studied countries. The results also revealed the negative effect of income, air pollution, and the number of doctors on infant mortality while positive on life expectancy.

Based on the positive impact of health expenditures on health outcomes, it is necessary that the government should facilitate the healthcare and overall health system by constantly supporting it through productive health spending and appropriate and timely policies. There is a need to strengthen the fundamentals of the health system and increase the number of medical staff like doctors. However, the increase in health expenditures in the OECD countries in recent years raises serious concerns about fiscal sustainability in the long run. Therefore, apart from health spending, the government has to focus on other measures like economic and environmental that ensure positive health outcomes. For this purpose, the government should work on by including patient voice while formulating health policy to obtain productive outcomes at the least cost. The OECD countries need to protect the quality of the environment. The deterioration in the environment increases the occurrence of diseases that enhances health spending. Therefore, the OECD countries should focus on promoting renewable energy consumption.

## Data availability statement

The datasets presented in this study can be found in online repositories. The names of the repository/repositories and accession number(s) can be found here: https://data.oecd.org/.

## Author contributions

AA: original idea, paper write-up, methodology, and data collection. SH: methodology, results, and paper write-up. NM: methodology and results. MY: methodology, results, and final validation.
